# Comparison of protective effect of ascorbic acid on redox and endocannabinoid systems interactions in *in vitro* cultured human skin fibroblasts exposed to UV radiation and hydrogen peroxide

**DOI:** 10.1007/s00403-017-1729-0

**Published:** 2017-03-11

**Authors:** Agnieszka Gęgotek, Katarzyna Bielawska, Michał Biernacki, Ilona Zaręba, Arkadiusz Surażyński, Elżbieta Skrzydlewska

**Affiliations:** 1 Department of Analytical Chemistry, Medical University of Bialystok, Mickiewicza 2D, 15-222 Bialystok, Poland; 2 Department of Medicinal Chemistry, Medical University of Bialystok, Bialystok, Poland

**Keywords:** Fibroblasts, Hydrogen peroxide, Nrf2, UV radiation, Ascorbic acid, Endocannabinoid system

## Abstract

The mechanisms of biological activity of commonly used natural compounds are constantly examined. Therefore, the aim of this study was to compare ascorbic acid efficacy in counteracting the consequences of UV and hydrogen peroxide treatment on lipid mediators and their regulative action on antioxidant abilities. Skin fibroblasts exposed to UVA and UVB irradiation, treated with hydrogen peroxide and ascorbic acid. The redox system was estimated through reactive oxygen species (ROS) generation (electron spin resonance spectrometer) and antioxidants level/activity (HPLC/spectrometry) which activity was evaluated by the level of phospholipid metabolites: 4-hydroxynonenal, malondialdehyde, 8-isoprostanes and endocannabinoids (GC/LC-MS) in the human skin fibroblasts. Protein and DNA oxidative modifications were also determined (LC). The expression of nuclear factor erythroid 2-related factor 2 (Nrf2), its activators and inhibitors as well as pro/anti-apoptotic proteins and endocannabinoid receptors was examined (Western blot) and collagen metabolism was evaluated by collagen biosynthesis and prolidase activity (spectrometry). UVA and UVB irradiation and hydrogen peroxide treatment enhanced activity of xanthine and NADPH oxidases resulting in ROS generation as well as diminution of antioxidant phospholipid protection (glutathione peroxidase-glutathione-vitamin E), what led to increased lipid peroxidation and decreased endocannabinoids level. Dysregulation of cannabinoid receptors expression and environment of transcription factor Nrf2 caused apoptosis induction. Ascorbic acid partially prevented ROS generation, antioxidant capacity diminution and endocannabinoid systems disturbances but only slightly protected macromolecules such as phospholipid, protein and DNA against oxidative modifications. However, ascorbic acid significantly prevented decrease in collagen type I biosynthesis. Ascorbic acid in similar degree prevents UV (UVA and UVB) and hydrogen peroxide-dependent redox imbalance. However, this antioxidant cannot efficiently protect cellular macromolecules and avert metabolic dysregulation leading to apoptosis.

## Introduction

The main responsibility of human skin is to protect the organism against physical and chemical factors, but it also functions in communication with the surrounding environment. However, the skin is exposed to a number of factors every day that may lead to disturbances in the metabolism of the cells comprising its different layers. The main group of cells that form the dermis are fibroblasts, which are responsible for the production of basic structural components of the skin, including collagen, elastin, and glycosaminoglycans, that appropriate the physical and mechanical properties of the skin. One of the main physical stress factors for the human skin is UV radiation in solar radiation.

The UV spectrum that reaches the earth’s surface contains UVB (280–320 nm) and UVA (320–400 nm) radiation. UVA and UVB radiation exhibit different biological effects; however, a common point of their activities is the enhanced generation of reactive oxygen species (ROS) [1, 2]. Moreover, UVA leads to the activation of endogenous photosensitizers, which in addition, enhance ROS generation [3]. However, fibroblast metabolism may also be affected by treatment with chemical oxidant such as hydrogen peroxide [4]. Both UV radiation and hydrogen peroxide impair fibroblasts mitochondrial antioxidant defence [5]. Imbalanced oxidative conditions are responsible for changes in phospholipids metabolism including polyunsaturated fatty acids (PUFAs) ROS-dependent peroxidation as well as enzymatic metabolism. Extremely reactive electrophiles are generated during ROS reactions with PUFAs, e.g., 4-hydroxyalkenals that having *α,β*-unsaturated bond can modify proteins, phospholipids and DNA [6]. Other lipid mediators, generated also during enhanced ROS level, are endocannabinoids belonging to endocannabinoid system. Previous data demonstrated that endocannabinoid system components are present within the mechanism that is regulated at transcriptional fibroblasts [7]. The most widely existing endocannabinoids are anandamide (AEA) and 2-arachidonoylglycerol (2-AG) that are synthesized on demand from arachidonic acid–containing phospholipid precursors and bind to G protein–coupled cannabinoid receptors (mainly CB1/2 but also VR1 and GPR55) [8]. It was indicated that CB1 activation is responsible for oxidative stress formation, while CB2 prevents ROS generation; however, both CB1 and CB2 stimulate the MAP kinase cascade and cause induction the pro-inflammatory cascade [9]. Moreover, it was earlier suggested that VR1 prevents neurons against oxidative stress and may play a protective role via inhibition of an inflammatory response [10]. Furthermore, UV radiation affects the degradation of collagen and elastin, and the consequence of these processes is premature aging of the skin [11, 12]. Since lipid mediators participate in cellular metabolic pathways changes in their level may lead to disturbances in antioxidant mechanisms and regulation of cell death.

The major determinant of cellular response to oxidative conditions is endogenous antioxidant defence as well as molecular level. It is known that electrophiles may act as secondary messengers and activate the nuclear transcription factor Nrf2 which under physiological conditions is biosynthesized permanently [13]. However, its level in the cytoplasm is regulated by the formation of the Nrf2-Keap1 complex [14]. It was shown that oxidative stress leads to Nrf2 activation through its phosphorylation and translocation to the nucleus where it binds to DNA and causes transcription of antioxidant protein genes [15, 16]. Data from the literature show that Nrf2 activation in human skin cells, following UVA radiation, acts in a cytoprotective manner by causing an enhanced transcription of antioxidant enzymes, e.g., glutathione peroxidase and heme oxygenase-1 [17]. Therefore, the Nrf2 response to external cells factors decides about molecular level of enzymatic antioxidants [18]. However, enzymatic as well as non-enzymatic antioxidants activity/level depends on lipid mediator electrophiles, in particular 4-HNE [19]. Moreover, it was also shown that Nrf2 fulfills an important function against apoptosis in dermal fibroblasts following UVA radiation [18]. Above data indicate that cross talk between redox and endocannabinoid systems in fibroblasts may be changed as a response for external factors activity.

To protect fibroblasts against damaging effects of physical and chemical external factors natural compound such ascorbic acid is often used. It participates in the main phospholipid protection mechanisms in the cell including glutathione (GSH) and vitamin E [20]. Ascorbic acid applied to the skin provides significant protection against erythema and sunburn cell formation [21]. Data from the literature indicate that ascorbic acid showed photoprotective potential and normalize MMP-1 mRNA and mitochondrial membrane polarization in UVA-irradiated human skin fibroblasts [22, 23]. Moreover, ascorbic acid in fibroblasts is essential for collagen biosynthesis as a cofactor for prolyl and lysyl hydroxylase and as a stimulus for collagen gene expression [11, 24]. However, there was not examined effect of ascorbic acid on lipid mediators and antioxidant defence mechanisms in fibroblasts after UV radiation as well as hydrogen peroxide treatment.

Therefore, the aim of this study was to compare the efficiency of ascorbic acid in preventing the formation of changes in the interactions between electrophiles and endocannabinoid system as a response to UV (including UVA and UVB) irradiation and hydrogen peroxide treatment in human skin fibroblasts.

## Materials and methods

### Cell culture and treatment

Human fibroblasts (CCD 1112Sk) were obtained from American Type Culture Collection. The cells were cultured in Dulbecco’s Modified Eagle Medium (DMEM) supplemented with foetal bovine serum (10%) and containing 50 U/ml penicillin and 50 μg/ml streptomycin. The cells were cultured in a humidified atmosphere of 5% CO_2_ at 37 °C. When the cells (passage 6–8) reached 70% confluence they were subjected to stress conditions.

One group of cells was washed with phosphate buffered saline (PBS) (37 °C) and exposed to UV radiation in cold PBS (4 °C) to avoid heat stress and oxidation of the medium components. The exposure dose was chosen corresponding to 70% cell viability, as measured by the MTT assay [25]. The cells were irradiated on ice at a distance of 15 cm from the six lamps (Bio-Link Crosslinker BLX 312/365; Vilber Lourmat, Germany) that were assembled to have 6 W each, which corresponds to 4.2 and 4.08 mW/cm^2^, respectively for UVA (365 nm) and UVB (312 nm). The total radiation doses were equal to 20 J/cm^2^ for UVA and 200 mJ/cm^2^ for UVB. After the irradiation, the cells were incubated for 24 h under standard conditions without rinsing.

A second group of cells was incubated for 24 h under standard conditions in medium containing 200 µM H_2_O_2_ (the concentration of which was chosen corresponding to 70% cell viability). The control cells were incubated in parallel without irradiation and hydrogen peroxide treatment. To examine the effect of ascorbic acid on the fibroblasts, all of the cell groups, control cells, cells after UV irradiation and cells after the hydrogen peroxide treatment were cultured in medium containing 100 µM ascorbic acid. After a 24 h incubation, all of the cells were washed with PBS, collected by scraping into cold PBS and centrifuged. The cells were then resuspended in PBS and subjected to three freeze/thaw cycles. The total protein content in the cell lysate was measured using a Bradford assay [26].

### Intracellular ROS generation

#### Determination of pro-oxidants enzymes activities

Xanthine oxidase (XO—EC1.17.3.2) activity was assessed by uric acid formation from xanthine by measuring the increase in absorbance at 290 nm, according to the method of Prajda and Weber [27]. One unit of XO was defined as the amount of enzyme required to release 1 μM of uric acid per minute. Analyses were performed in three independent experiments. Enzyme specific activities were calculated in microunits per milligram of protein and expressed as a percentage of the enzyme specific activity calculated from the control cells (56.16 ± 2.37 µU/mg protein).

NADPH oxidase (NOX—EC 1.6.3.1) activity was measured by luminescence assay using lucigenin as a luminophore. One unit of NOX activity was defined as the amount of enzyme required to release 1 nmol of O_2_^−^ per minute. Analyses were performed in three independent experiments. Enzyme specific activities were calculated in RLUs (Relative Luminescence Units) per milligram protein [28] and expressed as a percentage of the enzyme specific activity calculated from the control cells (159 ± 6.17 RLU/mg protein).

#### Determination of reactive oxygen species (ROS) generation

The generation of superoxide anions and total ROS was detected using an electron spin resonance (ESR) spectrometer e-scan (Noxygen GmbH/Bruker Biospin GmbH, Germany), where selective interaction of the spin probe 1-hydroxy-3-methoxy-carbonyl-2,2,5,5-tetrame-thylpyrrolidine (CMH) (200 µM) with ROS formed a stable nitroxide CM-radical with a half-life of 4 h. Thus, ROS formation was measured from the kinetics of nitroxide accumulation according to the electron spin resonance (ESR) amplitude of the low field component of the ESR spectra [29]. The rate of superoxide radical formation was determined by measuring superoxide dismutase (SOD)-inhibited nitroxide generation Analyses were performed in three independent experiments. The generation of ROS and superoxide anions were calculated as micromolar concentration per minute per milligram of protein and expressed as a percentage of the value determined from the control cells (0.095 ± 0.006 and 0.035 ± 0.002 µM/min/mg protein for ROS and superoxide anions, respectively).

### Antioxidant defence system

#### Determination of the antioxidant enzymes activities

Glutathione peroxidase (GSH-Px—EC.1.11.1.6) activity was assessed spectrophotometrically using the method of Paglia and Valentine [30]. GSH-Px activity was assayed by measuring the conversion of NADPH to NADP^+^. One unit of GSH-Px activity was defined as the amount of enzyme catalyzing the oxidation of 1 µmol NADPH per minute at 25 °C and pH 7.4. Analyses were performed in three independent experiments. Enzyme specific activity was calculated in milliunits per milligram of protein and expressed as a percentage of the enzyme specific activity determined from the control cells (9.95 ± 0.56 mU/mg protein).

Glutathione reductase (GSSG-R—EC.1.6.4.2) activity was measured according to the method of Mize and Longdon [31] by monitoring the oxidation of NADPH at 340 nm at a pH 7.4. Enzyme activity is expressed in units per milligram of protein. One unit of GSSG-R oxidized 1 mmol of NADPH/min at 25 °C and pH 7.4. Analyses were performed in three independent experiments. Enzyme specific activity was calculated in milliunits per milligram of protein and expressed as a percentage of the enzyme specific activity determined from the control cells (27.3 ± 1.9 mU/mg protein).

Superoxide dismutase (Cu/Zn–SOD—EC.1.15.1.1) activity was determined according to the method of Misra and Fridovich [32] as modified by Sykes [33], which measures the activity of cytosolic SOD. One unit of SOD was defined as the amount of enzyme, which inhibits epinephrine oxidation to adrenochrome by 50%. Analyses were performed in three independent experiments. Enzyme specific activity was calculated in milliunits per milligram of protein and expressed as a percentage of the enzyme specific activity determined from the control cells (26.1 ± 2.1 mU/mg protein).

#### Determination of the non-enzymatic antioxidants level

Glutathione was quantified using the capillary electrophoresis (CE) method of Maeso [34]. Samples were sonicated in Eppendorf tubes with 2 ml of a mixture containing ACN/H_2_O (62.5:37.5, v/v) and centrifuged at 29,620*g* for 10 min. The supernatants were immediately measured by CE. Separation was performed on a 47 cm capillary (40 cm effective length) and 50 m i.d. and was operated at 27 kV with UV detection at 200 ± 10 nm. Analyses were performed in three independent experiments. The GSH concentration was determined using a calibration curve range of 1–120 nmol/L (*r* ^2^ = 0.9985) and normalized for milligrams of protein. GSH concentrations are expressed as a percentage of the GSH concentration found in the control cells (12.61 ± 0.91 nmol/mg protein).

High-performance liquid chromatography (HPLC) was used to detect the level of vitamin C [35], A and E [36]. Briefly, the cell lysates were centrifuged (1000×*g*, 10 min). For the determination of vitamin C, the supernatants were mixed with an equal volume of metaphosphoric acid. The separation was performed using an RP-18 column and UV detection at 250 nm. The mobile phase was phosphate buffer (pH 2.8) and water (97:3). The flow rate was 0.7 ml/min. Vitamins A and E were extracted from the cell lysates using hexane. The hexane phase was removed, dried and diluted in ethanol, and 50 µl of the mixture was injected on the column. UV detection at 294 nm was applied. The flow rate was 1 ml/min of methanol and water (95:5). The concentration of the vitamins was determined using a calibration curve range of 1.25 to 20 µg/mL (*r* ^2^ = 0.9999) for vitamin C, 0.125 to 1 mg/L (*r* ^2^ = 0.9998) for vitamin A, and 5 to 25 mg/L (*r* ^2^ = 0.9999) for vitamin E. Analyses were performed in three independent experiments. The vitamins concentration was normalized for milligrams of protein and expressed as a percentage of vitamins concentration found in the control cells (415 ± 22, 241 ± 13, and 1.19 ± 0.06 µg/mg protein for vitamin C, A and E respectively).

### Lipid mediators

#### Determination of lipid peroxidation products

Lipid peroxidation was estimated by measuring the levels of 4-hydroxynonenal (4-HNE) and malondialdelhyde (MDA). Aldehydes were measured by GC/MSMS, as the *O*-(2,3,4,5,6-pentafluoro-benzyl)-oxime-trimethylsilyl (*O*-PFB-oxime-TMS) derivatives, using the modified method of Luo [37]. Benzaldehyde-D_6_ was added as an internal standard to the cell lysates, and aldehydes were derivatized by the addition of *O*-(2,3,4,5,6-pentafluoro-benzyl) hydroxylamine hydrochloride (0.05 M in PIPES buffer, 200 µL) and incubation for 60 min at room temperature. After incubation, samples were deproteinized by the addition of 1 mL of methanol, and *O*-PFB-oxime aldehyde derivatives were extracted by the addition of 2 mL of hexane. The top hexane layer was transferred into borosilicate tubes and evaporated under a stream of argon gas, followed by the addition of *N,O*-bis(trimethylsilyl)trifluoroacetamide in 1% trimethylchlorosilane. A 1 µL aliquot was injected onto the column. Derivatized aldehydes were analyzed using a 7890 A GC—7000 quadrupole MS/MS (Agilent Technologies, Palo Alto, CA) equipped with a HP-5 ms capillary column (0.25 mm internal diameter, 0.25 µm film thickness, 30 m length). Derivatized aldehydes were detected in selected ion monitoring (SIM) mode. The ions used were as follows: *m*/*z* 333.0 and 181.0 for 4-HNE-PFB-TMS, *m*/*z* 204.0 and 178.0 for MDA-PFB. The LOD were as follows: 4 pmol/mL for 4-HNE and 6 pmol/mL for MDA. Analyses were performed in three independent experiments. Obtained results were normalized for milligrams of protein. 4-HNE and MDA concentrations are expressed as a percentage of the values determined for control cells (56.7 ± 3.7 and 178 ± 15 nmol/mg protein for 4-HNE and MDA, respectively).

8-Iso-prostaglandin F2*α* (8-isoPGF2*α*) was assayed by the modified LC-MS method of Coolen [38] using an Agilent 1290 UPLC system interfaced with an Agilent 6460 triple quadrupole mass spectrometer with an electrospray ionization source (ESI). Briefly, samples were purified using a SEP-PAK C18 column containing octadecylsilyl silica gel. A reverse phase C18 column (2.1 × 150, 3.5 mm) was employed. The separation was performed using a linear gradient of water (pH 5.7) and acetonitrile. 8-isoPGF2*α*–d_4_ was used as an internal standard. 8-isoPGF2*α* was analyzed in negative-ion mode using MRM mode: m/z 353.2→193.1 (for 8-isoPGF2*α*) and 357.2→197.1 (for 8-isoPGF2 *α*-d_4_). The limit of detection (LOD) was 1 pg/mL. Analyses were performed in three independent experiments. Obtained results were normalized for milligrams of protein. 8-isoPGF2*α* concentrations are expressed as a percentage of the concentration determined for control cells (6.6 ± 0.4 pg/mg protein).

#### Determination of endocannabinoids

Anandamide (AEA) and 2-arachidonoylglycerol (2-AG) were quantified using modified ultrahigh performance liquid chromatography-tandem mass spectrometry (UPLC-MS/MS) by the Lam method [39]. Octadeuterated endocannabinoids AEA-d_8_ and 2-AG-d_8_ were added as internal standards to the cell lysates, and all cannabinoids were isolated using solid phase extraction (SPE). UPLC–MS/MS analysis was performed using an Agilent 1290 UPLC system with a Zorbax Extend C18 column (2.1 × 150, 1.8 mm, Agilent, Santa Clara, CA, USA) and interfaced with an Agilent 6460 triple quadrupole mass spectrometer with an electrospray ionization source (ESI). The samples were analyzed in positive-ion mode using multiple reaction monitoring (MRM). Transition of the precursor to the product ion was as follows: *m*/*z* 348.3→62.1 for AEA; *m*/*z* 379.3→287.2 for 2-AG. The LODs were as follows: 2 pg/mL for AEA and 40 pg/mL for 2-AG. Analyses were performed in three independent experiments. Obtained results were normalized for milligrams of protein. Endocannabinoids concentrations are expressed as a percentage of the concentrations found in control cells (15.9 ± 0.7 and 238 ± 16 fmol/mg protein for AEA and 2-AG, respectively).

### DNA modifications

#### Determination of 8-hydroxy-2′-deoxyguanosine

8-hydroxy-2′-deoxyguanosine (8-OHdG) was assayed by the modified LC-MS method of Dizdaroglu [40]. DNA isolation was performed using a commercial kit (GenElute Mammalian Genomic DNA Miniprep Kit, Sigma; USA). The DNA concentrations in the preparations were determined spectrophotometrically, and samples were stored at −70 °C until hydrolysis. DNA hydrolysis into individual nucleosides was achieved by mixing DNA samples (200 μl) with 100 μl of 40 mM sodium acetate/0.1 mM ZnCl_2_ (pH 5.1) and 20 μl of nuclease P1 solution (20 μg protein). Samples were incubated for 1 h at 37 °C. Thereafter, 30 μl of 1 M Tris–HCl (pH 7.4) and 5 μl of alkaline phosphatase solution containing 1.5 units of the enzyme were added to each sample following 1 h incubation at 37 °C. All DNA hydrolysates were ultrafiltered using Ultrafree-MC filter units (cut-off of 5000 Da). 8-OHdG concentrations in hydrolysates were determined using an Agilent 1290 LC system and an Agilent 6460 triple quadrupole mass spectrometer equipped with an electrospray ionization ESI source. Solvent A (0.1% formic acid in water) and solvent B (0.1% formic acid in methanol) were used in gradient mode to achieve the desired sample separations. The flow rate was set at 0.4 ml/min while the following gradient was run: 0 min, 5% solvent B; 0–8.0 min, 50% solvent B; 8.0–8.1 min, 100% solvent B; 8.01–12.0 min, 100% solvent B; 12.0–13.0 min, 5% solvent B. LC–MS/MS analysis was performed using an Agilent 1290 HPLC system interfaced with an Agilent 6560 triple quadrupole mass spectrometer with an electrospray ion source (ESI). The samples were analyzed in the positive ion multiple reaction monitoring (MRM) mode and the transitions of the precursors to the product ions were as follows: *m*/*z* 284.1→168 (quantifier ion) and 284.1→69 (qualifier ion). The concentrations of 8-OHdG in the samples were calculated using a calibration curve range of 10–1000 pg/ml (*r* ^2^ = 0.9995), which was normalized for milligrams of DNA. Analyses were performed in three independent experiments. 8-OHdG levels are expressed as a percentage of the 8-OHdG concentration determined in control cells (7.48 ± 0.49 ng/mg DNA).

### Protein modifications

#### Determination of structure modification

Protein oxidative modifications were estimated according to the levels of carbonyl groups and dityrosine. The carbonyl groups were determined spectrophotometrically (370 nm) using 2,4-dinitrophenylhydrazine [41]. The concentrations of carbonyl groups in the samples were calculated using a calibration curve (0.2–2 mmol/L, *r* ^2^ = 0.9988). To detect dityrosine samples were diluted in 0.1 mol/L H_2_SO_4_ (1:10), and fluorescence emission/excitation at 325 nm/420 nm was measured. The results were normalized to fluorescence of 0.1 mg/mL quinine sulfate in 0.1 mol/L H_2_SO_4_ (Ex_325nm_/Em_420nm_ = 405), which is equivalent to 1 U of dityrosine. Analyses were performed in three independent experiments. The results were normalized for milligrams of protein and are expressed as a percentage of the values obtained for control cells (0.37 ± 0.04 and 0.41 ± 0.04 U/mg protein for carbonyl groups and dityrosine, respectively).

#### Determination of protein expression

Western blot analysis of cellular proteins was performed according to Eissa and Seada [42]. Each analysis was performed in three independent experiments. Whole cell lysates or membrane fractions were mixed with sample loading buffer (Laemmle buffer containing 5% 2-mercaptoethanol), heated at 95 °C for 10 min, and separated by 10% Tris–glycine SDS–PAGE. The same procedure was used to prepare the negative control (containing pure PBS buffer) and the positive control (commercially purchased complete cell lysate—Santa Cruz Biotechnology, Santa Cruz, CA, USA). As internal loading controls, *β*-actin and Na^+^/K^+^ ATPase (for cell lysates and membrane fractions, respectively) were used. Separated proteins in the gels were electrophoretically transferred onto nitrocellulose membranes. The blotted membranes were blocked with 5% skim milk in TBS-T buffer (5% Tween 20) for 1 h. Primary antibodies were raised against Nrf2, phospho-Nrf2 (pSer40), Keap1, HO-1, Bcl-2, cyt c, p53, ERK1/2, GPR55, collagen type I, *β*-actin, and Na^+^/K^+^ ATPase were purchased from Sigma–Aldrich (St. Louis, MO, USA) and used at a concentration of 1:1000. Bach1, KAP1, p21, p62, NF*κ*B(p52), TNF*α*, CB1, CB2, VR1, and caspases 3, 8, and 9, purchased from Santa Cruz Biotechnology (Santa Cruz, CA, USA), were also used at a concentration of 1:1000. Protein bands were visualized using the BCIP/NBT liquid substrate system (Sigma–Aldrich, St. Louis, MO, USA) and quantitated using the Versa Doc System and Quantity One software (Bio-Rad Laboratories Inc., CA). The results are expressed as a percentage of the expression determined in control cells.

#### Determination of protein localisation

Cells were seeded in BD Falcon™ 96-well black, clear-bottom tissue culture plates at 10,000 cells per well. These plates are optimized for imaging applications. Analysis were performed in three independent experiments. After incubation, cells were rinsed with PBS and fixed with a 3.7% formaldehyde solution at room temperature for 10 min. Cells were then washed three times with PBS and permeabilized with 0.1% Triton X-100 at room temperature for 5 min. Next, the cells were washed twice with PBS, and non-specific binding was blocked by incubation in 3% FBS at room temperature for 30 min. The cells were rinsed and incubated with either anti-Nrf2 rabbit polyclonal antibodies (Sigma–Aldrich, St. Louis, MO, USA; 1:1000) or anti-NF*κ*B (p52) mouse polyclonal antibodies (Santa Cruz Biotechnology, Santa Cruz, CA, USA) for 1 h at room temperature. Cells were then washed three times with PBS and incubated with FITC-conjugated anti-rabbit secondary antibodies (BD Pharmingen, San Diego, CA) for 60 min in the dark. After washing, nuclei were stained with Hoechst 33,342 (2 µg/ml) and analyzed using a BD Pathway 855 confocal microscope with a 40 × (0.75 NA) objective. The cytoplasmic and nuclear fluorescence intensities of stained cells were analyzed, and images of FITC-labeled cells were acquired using a 488/10 excitation laser and a 515LP emission laser.

#### Determination of collagen synthesis

The incorporation of the radioactive precursor into the proteins was measured after labelling the confluent cells in serum-free medium for 24 h with the 5-[^3^H] proline (5 μCi/ml, 28 Ci/mmol). The incorporation of the label into collagen was determined by digesting the proteins with purified *Clostridium histolyticum* collagenase according to the method of Peterkofsky [43]. Analyses were performed in three independent experiments. The results are shown as combined values for the cells plus the medium fractions and are expressed as a percentage of the control cells.

#### Determination of prolidase activity

The activity of prolidase was determined according to the method of Myara [44], which is based on the colorimetric determination of proline using Chinard’s reagent. The cells were scraped off and centrifuged at 200×*g* for 15 min, and the supernatant was discarded. The cell pellet was suspended in 1 ml of 50 mM HEPES, pH 7.8, and sonicated for 3 × 10 s at 0 °C. The samples were then centrifuged (12,000×*g*, 30 min) at 4 °C, and the supernatant was used for protein determination. The activation of prolidase requires an incubation with Mn(II), where 100 μl of the cell extract supernatant was mixed with 100 μl of 50 mM HEPES, pH 7.8, containing MnCl_2_ at a final concentration of 1 mM in the mixture. After incubating for 24 h at 37 °C, the prolidase reaction was initiated by adding 100 μl of the activated mixture to 100 μl of 94 mM glycyl-proline (Gly-Pro) for a final concentration of 47 mM. After an additional incubation for 1 h at 37 °C, the reaction was terminated by the addition of 1 ml of 0.45 M trichloroacetic acid. To parallel the blank tubes, trichloroacetic acid was added at time “zero.” The samples were centrifuged at 10,000×*g* for 15 min. The released proline was determined by adding 0.5 ml of the trichloroacetic acid supernatant to 2 ml of a 1:1 mixture of glacial acetic acid: Chinard’s reagent (25 g of ninhydrin dissolved at 70 °C in 600 ml of glacial acetic acid and 400 ml of 6 M orthophosphoric acid) and incubated for 10 min at 90 °C. The amount of proline released was determined colorimetrically by monitoring the absorbance at 515 nm and calculated using the proline standards. Analyses were performed in three independent experiments. The enzyme activity is reported in nanomoles of proline released per minute per milligram of protein and is expressed as a percentage of the prolidase activity determined in control cells (40 ± 4 nmol/min/mg protein).

### Statistical analysis

Data were analyzed using standard statistical analyses, including one-way Student’s test for multiple comparisons, and the results are expressed as the mean ± standard deviation (SD) for *n* = 3. *P* values less than 0.05 were considered significant.

## Results

### Inflammatory and oxidative processes

**Fig. 1:**
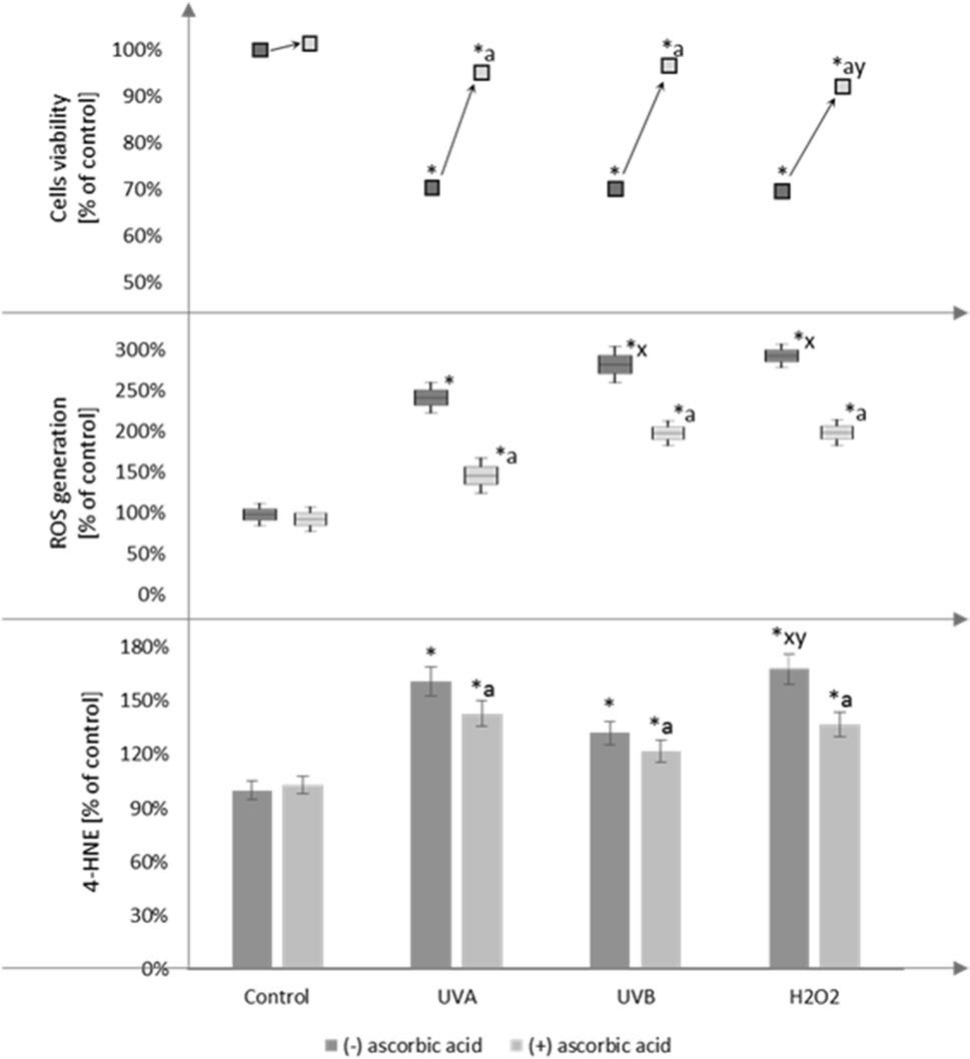
The comparison of cell viability, ROS generation, and 4-HNE level in fibroblasts after exposure to UVA (20 J/cm^2^), UVB radiation (200 mJ/cm^2^), H_2_O_2_ (200 µM), and ascorbic acid (100 µM) expressed as a percentage of the value of the control cells. Mean values ± SD of three independent experiments are presented. *Asterisk* statistically significant differences vs. control group, *p* < 0.05; *a* statistically significant differences vs. group without ascorbic acid, *p* < 0.05; *x* statistically significant differences vs. UVA group, *p* < 0.05; *y* statistically significant differences vs. UVB group, *p* < 0.05

**Fig. 2:**
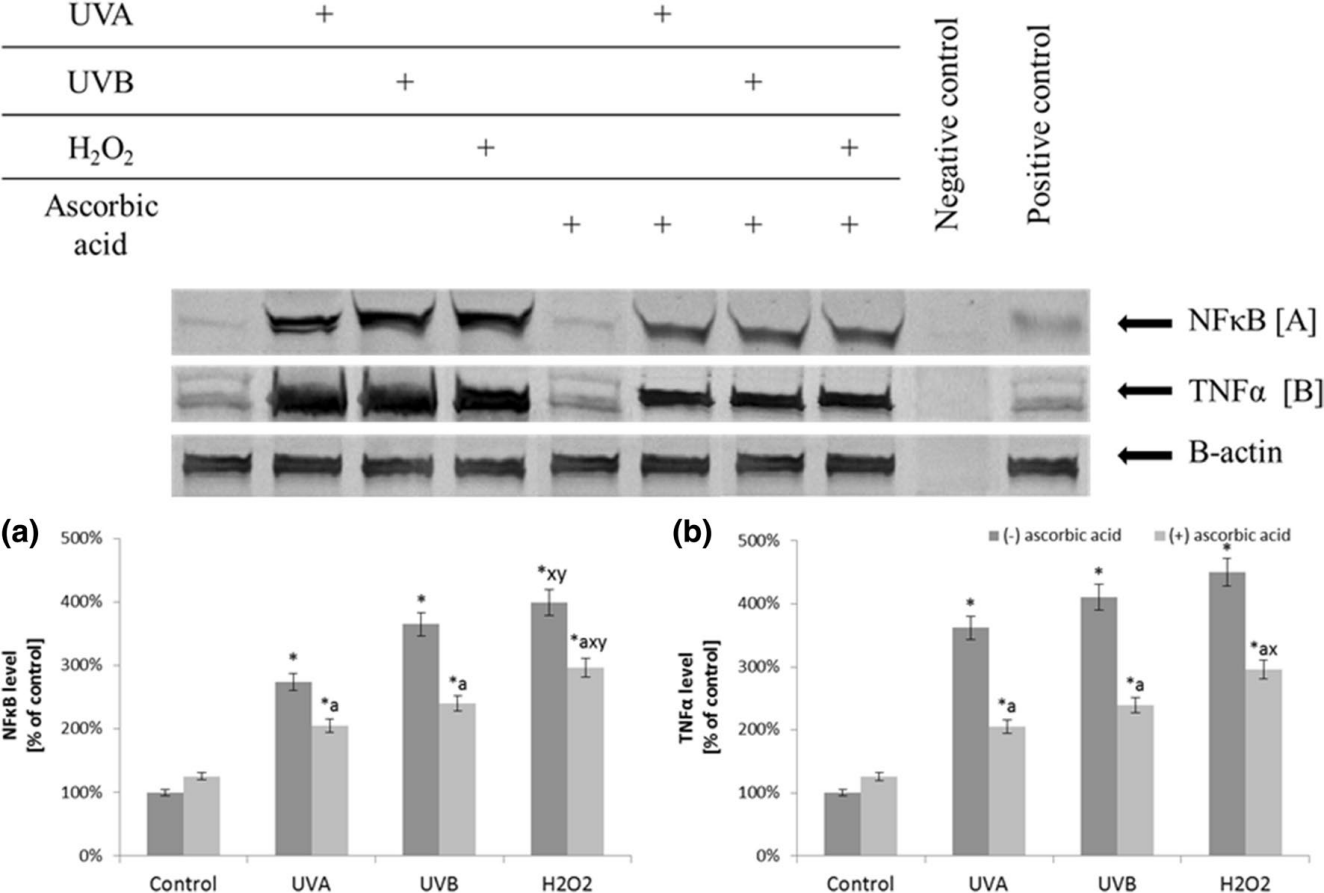
The level of transcription factor NF*κ*B and its target protein—TNF*α* in fibroblasts after exposure to UVA (20 J/cm^2^), UVB radiation (200 mJ/cm^2^), H_2_O_2_ (200 µM), and ascorbic acid (100 µM) expressed as a percentage of the value of the control cells. Mean values ± SD of three independent experiments are presented. *Asterisk* statistically significant differences vs. control group, *p* < 0.05; *a* statistically significant differences vs. group without ascorbic acid, *p* < 0.05; *x* statistically significant differences vs. UVA group, *p* < 0.05; *y* statistically significant differences vs. UVB group, *p* < 0.05

Ascorbic acid reduced the UV radiation and H_2_O_2_ induced expression of pro-inflammatory mediators and prevents intracellular oxidative processes, as well as enhances viability of fibroblasts (Fig. 1). The supplementation of the cells with ascorbic acid reduced the NF*κ*B levels (by approximately 25% for the cells treated with H_2_O_2_ and exposed to UVA radiation and by approximately 35% for the cells exposed to UVB radiation) and TNF*α* levels (by approximately 55% for all conditions compared to cells not treated with ascorbic acid) (Fig. 2).

**Fig. 3:**
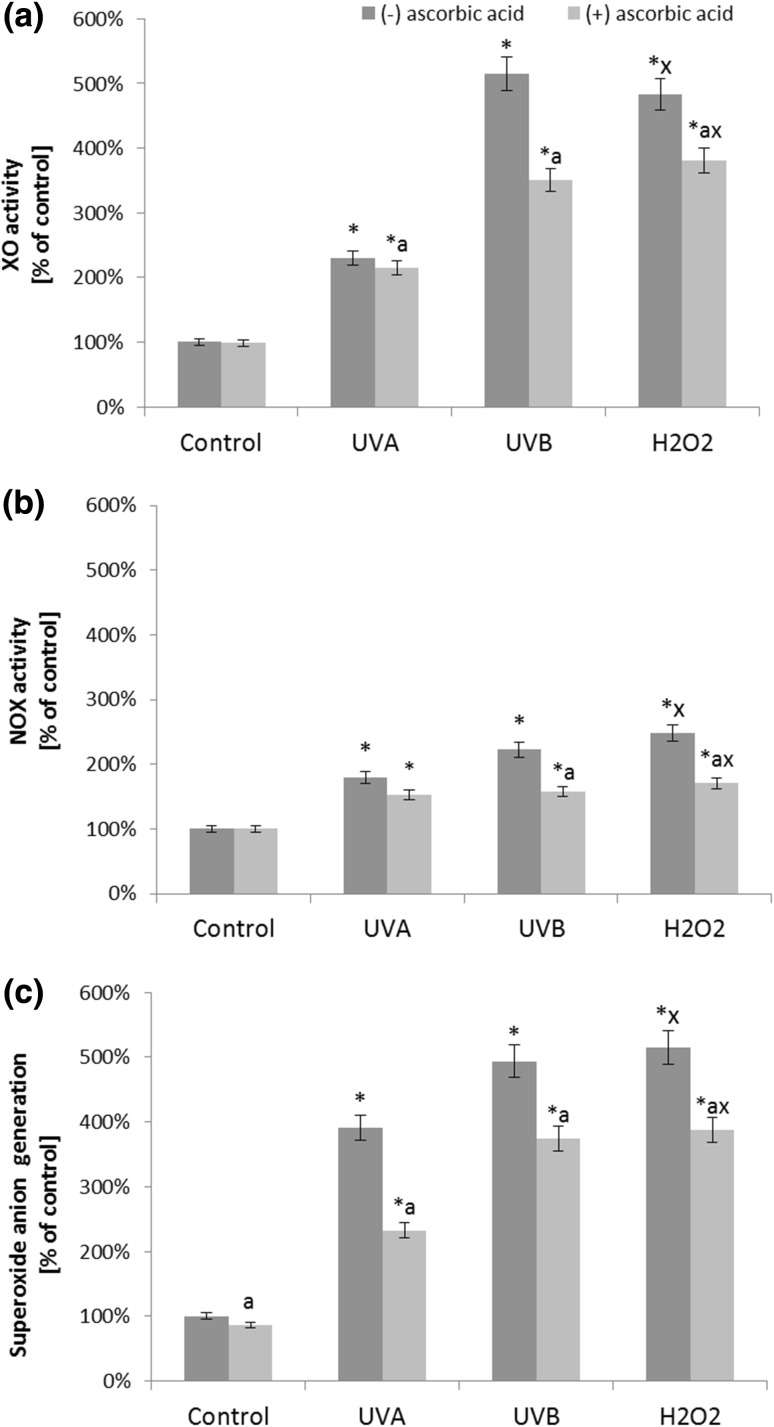
The xanthine oxidase (**a**) and NADPH oxidase (**b**) activity, and superoxide anion generation (**c**) in fibroblasts after exposure to UVA (20 J/cm^2^), UVB radiation (200 mJ/cm^2^), H_2_O_2_ (200 µM), and ascorbic acid (100 µM) expressed as a percentage of the value of the control cells. Mean values ± SD of three independent experiments are presented. *Asterisk* statistically significant differences vs. control group, *p* < 0.05; *a* statistically significant differences vs. group without ascorbic acid, *p* < 0.05; *x* statistically significant differences vs. UVA group, *p* < 0.05; *y* statistically significant differences vs. UVB group, *p* < 0.05

Treatment of fibroblasts with ascorbic acid, after UV irradiation, as well as H_2_O_2_ treatment, leads to lower changes in the levels/activities of oxidants and antioxidants. The results show that fibroblasts irradiated with UVA and UVB and treated with H_2_O_2_ at the tested doses are characterized by a higher activity of xanthine oxidase, which is an enzyme that is responsible for superoxide anion generation, in comparison with the control cells. Cells treatment with ascorbic acid, after UV, particularly UVB irradiation or hydrogen peroxide, results in a reduction of XO activity by approximately 30 and 20%, respectively (Fig. 3). Furthermore, ascorbic acid reduced the activity of another superoxide anion generating enzyme, NADPH oxidase by approximately 20–30% compared to cells without supplementation (Fig. 3).

Our results also show that in all cases, the ascorbic acid reduced total ROS as well as superoxide anion generation enhanced by UV irradiation. We found that supplementation of the medium with ascorbic acid, following UVA and UVB irradiation causes a decrease in total ROS up to 40%, and after treatment with H_2_O_2_—35% compared with the respective control groups of fibroblasts (Fig. 1). In the case of superoxide anion generation ascorbic acid causes a decrease up to 55% compared with the respective control groups of fibroblasts following UVB irradiation and after treatment with H_2_O_2_. Supplementation with ascorbic acid of the control fibroblasts and those irradiated with UVA causes reduction in superoxide anion generation by approximately 13 and 16%, respectively (Fig. 3).

### Antioxidant defence system

Ascorbic acid also prevents against UV radiation and hydrogen peroxide induced changes in the activity of antioxidant enzymes, as well as in the non-enzymatic antioxidants levels, in fibroblasts (Table 2). In all cases, supplementation with ascorbic acid leads to a reduction in GSH-Px activity by approximately 40% compared to fibroblasts not treated with ascorbic acid (Table 1). On the other hand, the addition of ascorbic acid to those cells increases Cu/Zn-SOD activity in fibroblasts subjected to UVA, UVB, and H_2_O_2_ by 9, 50 and 21%, respectively, compared to control groups of fibroblasts (Table 1). In addition, ascorbic acid supplementation to the fibroblasts treated with UVA, UVB, and H_2_O_2_ enhances the GSH levels by approximately 15, 10, and 20%, respectively, compared to fibroblasts not treated with ascorbic acid (Table 1). UVA, UVB, and H_2_O_2_ treatment decreased in the level of ascorbic acid in the cells (by approximately 24, 16, and 21, respectively). Likewise, in this case, ascorbic acid supplementation in the cells increases its cytoplasmic level by approximately 75, 51 and 103 for UVA, UVB, and H_2_O_2_, respectively, compared to fibroblasts without supplementation (Table 1).

**Table 1:** The activity of enzymatic (GSH-Px, GSSG-R, Cu,Zn-SOD) and the level of non-enzymatic (GSH, vitamins E and A) antioxidants in fibroblasts after exposure to UVA (20 J/cm^2^), UVB radiation (200 mJ/cm^2^), H_2_O_2_ (200 µM), and ascorbic acid (100 µM) expressed as a percentage of the value of the control cells

	Ascorbic acid	Control (%)	UVA (%)	UVB (%)	H_2_O_**2**_ (%)
Cu,Zn-SOD	−	100 ± 8	83 ± 4*	54 ± 3*	60 ± 3*^,x^
	+	102 ± 5	91 ± 4*^,a^	81 ± 4*^,a^	73 ± 4*^,a,x,y^
GSH-Px	−	100 ± 6	153 ± 11*	198 ± 12*	219 ± 10*^,x,y^
	+	99 ± 3*^,a^	132 ± 6*^,a^	155 ± 7*^,a^	134 ± 6*^,a,y^
GSSG-R	−	100 ± 7	178 ± 10*	205 ± 13	167 ± 8*^,x,y^
	+	108 ± 5	119 ± 6*^,a^	156 ± 8*^,a^	152 ± 8*^,a,x^
GSH	−	100 ± 7	64 ± 4*	59 ± 3*	55 ± 3*^,x^
	+	104 ± 4*^,a^	74 ± 3*^,a^	75 ± 3*^,a^	77 ± 3*^,a,x^
Vitamin E	−	100 ± 5	88 ± 3*	74 ± 4*	69 ± 3*^,x^
	+	102 ± 5	92 ± 3*	89 ± 4*	90 ± 2*^,x^
Vitamin A	−	100 ± 5	92 ± 1*	86 ± 1*	76 ± 1*^,x,y^
	+	102 ± 5	92 ± 1*^,a^	92 ± 1*^,a^	88 ± 3*^,a^
Vitamin C	−	100 ± 5	76 ± 4*	84 ± 4*	79 ± 4*^,y^
	+	155 ± 8*^,a^	133 ± 7*^,a^	172 ± 9*^,a^	160 ± 8*^a,x,y^

Mean values ± SD of three independent experiments are presented

*Statistically significant differences vs. control group, *p* < 0.05

^a^Statistically significant differences vs. group without ascorbic acid, *p* < 0.05

^x^Statistically significant differences vs. UVA group, *p* < 0.05

^y^Statistically significant differences vs. UVB group, *p* < 0.05

A similar direction of changes after UVA, UVB, and H_2_O_2_ treatment is observed in the case of the lipophilic antioxidants vitamins E and A (Table 1). However, there were no significant changes in vitamin E level after supplementation with ascorbic acid in all of the tested groups of fibroblasts. In the case of vitamin A levels, a ascorbic acid supplementation led to an approximate 10% increase in the vitamin A levels in the fibroblasts following UVA irradiation. Additionally, in the cases of H_2_O_2_ treatment, supplementation with ascorbic acid increased the vitamin A levels by approximately 12% (Table 1).

**Fig. 4:**
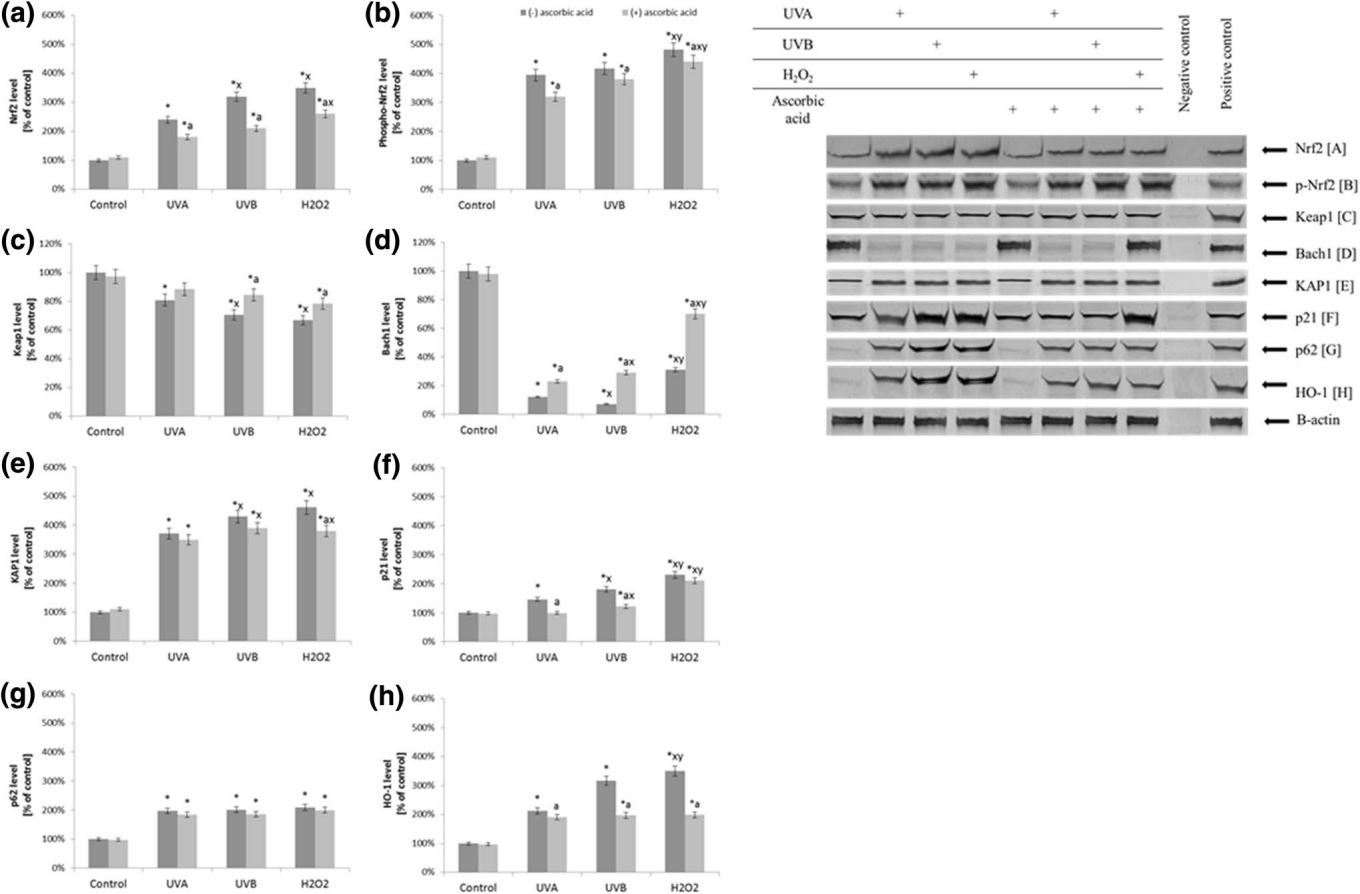
The level of Nrf2 (**a**), its phosphorylated form (pSer40) (**b**), its inhibitors: Keap1, Bach1 (**c, d**), and activators: KAP1, p21, p62 (**e, f, g**), and HO-1 (**h**) in fibroblasts after exposure to UVA (20 J/cm^2^), UVB radiation (200 mJ/cm^2^), H_2_O_2_ (200 µM), and ascorbic acid (100 µM) expressed as a percentage of the value of the control cells. Mean values ± SD of three independent experiments are presented. *Asterisk* statistically significant differences vs. control group, *p* < 0.05; *a* statistically significant differences vs. group without ascorbic acid, *p* < 0.05; *x* statistically significant differences vs. UVA group, *p* < 0.05; *y* statistically significant differences vs. UVB group, *p* < 0.05

**Fig. 5:**
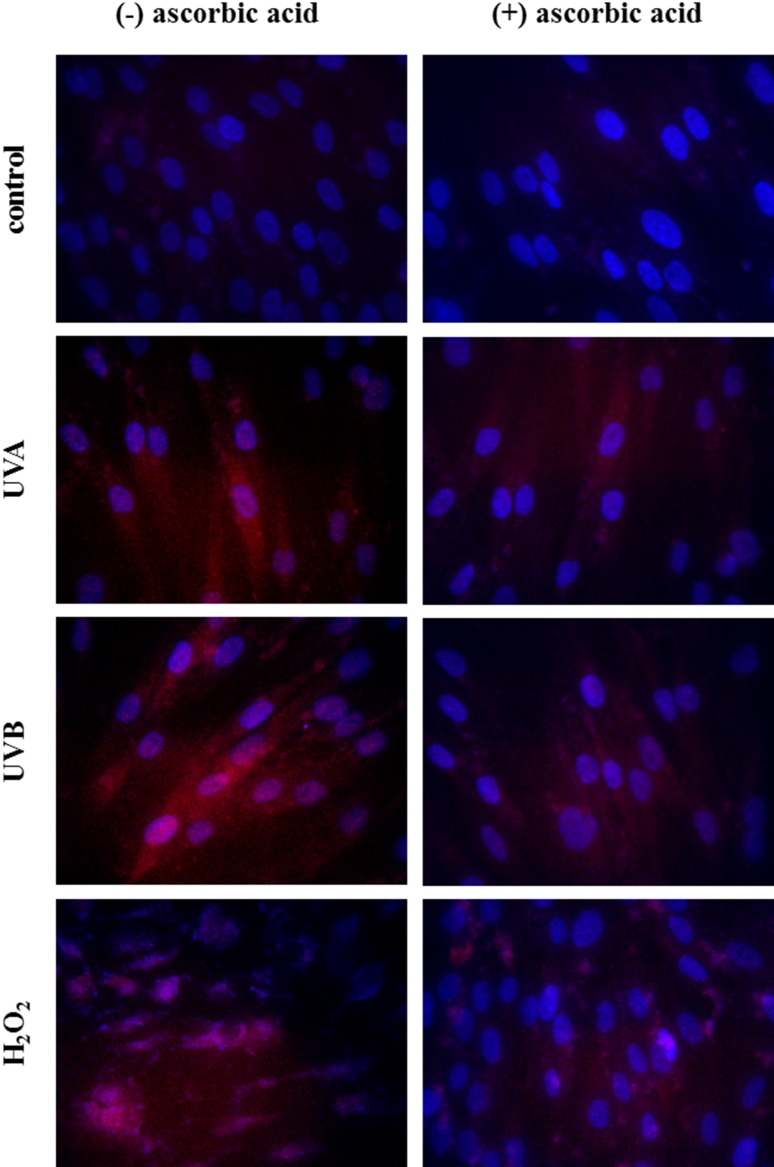
The cytoplasmic and nucleus level of Nrf2 in fibroblasts control cells and after exposure of UVA (20 J/cm^2^), UVB radiation (200 mJ/cm^2^), H_2_O_2_ (200 µM), and ascorbic acid (100 µM) (*blue* nucleus; *red* Nrf2)

Independent of the changes in the activities/levels of the antioxidants in the fibroblasts supplemented with ascorbic acid after UV radiation and H_2_O_2_ treatment, changes at the transcription level were observed. Ascorbic acid decreased the phosphorylated Nrf2 levels by approximately 20% compared to the fibroblasts not treated with ascorbic acid in the case of UVA irradiation and decreased them by 10% after UVB irradiation and H_2_O_2_ treatment (Fig. 4). Simultaneously, ascorbic acid reduced the Nrf2 translocation from the cytoplasm to the nucleus (Fig. 5) and cause significantly decreased in Nrf2 activating protein—KAP1 expression by 10 and 20% in the case of UVB radiation and H_2_O_2_ treatment, respectively. Ascorbic acid treatment also decrease the level of another Nrf2 activator—p21 by 10% in the case of UVA and UVB radiation. Moreover, ascorbic acid even two times increased Bach1 level in cells exposed to UVA radiation and more than three times in cells exposed to UVB radiation and treated with H_2_O_2_. Ascorbic acid also reduced the HO-1 levels by approximately 10% after UVA irradiation and by 40% after UVB irradiation and H_2_O_2_ treatment (Fig. 4).

### Lipid mediators

Enhanced ROS generation, as well as disruption of the antioxidant system, after UV radiation, and H_2_O_2_ treatment, result in enhanced oxidative modifications of lipids and proteins. Supplementation with ascorbic acid reduced the lipid peroxidation marker—MDA level by approximately 15, 30, and 60% in the fibroblasts following UVA, UVB, and H_2_O_2_ treatment, respectively (Table 2). Moreover, ascorbic acid reduced other lipid peroxidation products by approximately 10 and 20% the 4-HNE levels, and by approximately 20 and 10% the 8isoPGF2*α* levels in the fibroblasts following UVA and H_2_O_2_ treatment, respectively (Fig. 1; Table 2).

**Table 2:** The level of oxidative modification of lipid (8-isoPGF2*α*, MDA, 4-HNE), protein (carbonyl groups, dityrosine), and DNA (8-OHdG) in fibroblasts after exposure to UVA (20 J/cm^2^), UVB radiation (200 mJ/cm^2^), H_2_O_2_ (200 µM), and ascorbic acid (100 µM) expressed as a percentage of the value of the control cells

	Ascorbic acid	Control (%)	UVA (%)	UVB (%)	H_2_O_2_ (%)
8-isoPGF2*α*	−	100 ± 6	223 ± 11*	248 ± 12*^,x^	334 ± 17*^,x,y^
	+	94 ± 5	181 ± 9*^,a^	245 ± 12*^,x^	305 ± 15*^,a,x,y^
MDA	−	100 ± 8	150 ± 8*	136 ± 7*	204 ± 10*^,x,y^
	+	99 ± 5	126 ± 6*^,a^	129 ± 5^a,x^	182 ± 4*^,a,x,y^
4-HNE	−	100 ± 6	161 ± 8*	132 ± 7*	168 ± 8*^,x,y^
	+	103 ± 5	143 ± 7*^,a^	122 ± 7*^,a^	137 ± 7*^,a^
Carbonyl groups	−	100 ± 10	153 ± 8*	191 ± 10*^,x^	209 ± 10*
	+	109 ± 5	131 ± 7*^,a^	155 ± 8*^,a,x^	166 ± 8*^,a,x^
Dityrosine	−	100 ± 9	165 ± 8*	149 ± 7*	192 ± 10*^,x,y^
	+	101 ± 5	157 ± 8*	132 ± 7*^,a,x^	168 ± 8*^,a,y^
8-OHdG	−	100 ± 6	128 ± 6*	167 ± 8*^,x^	207 ± 9*^,x,y^
	+	101 ± 5	126 ± 6*	160 ± 7*^,x^	195 ± 9*^,a,x,y^

Mean values ± SD of three independent experiments are presented

*Statistically significant differences vs. control group, *p* < 0.05

^a^Statistically significant differences vs. group without ascorbic acid, *p* < 0.05

^x^Statistically significant differences vs. UVA group, *p* < 0.05

^y^Statistically significant differences vs. UVB group, *p* < 0.05

**Fig. 6:**
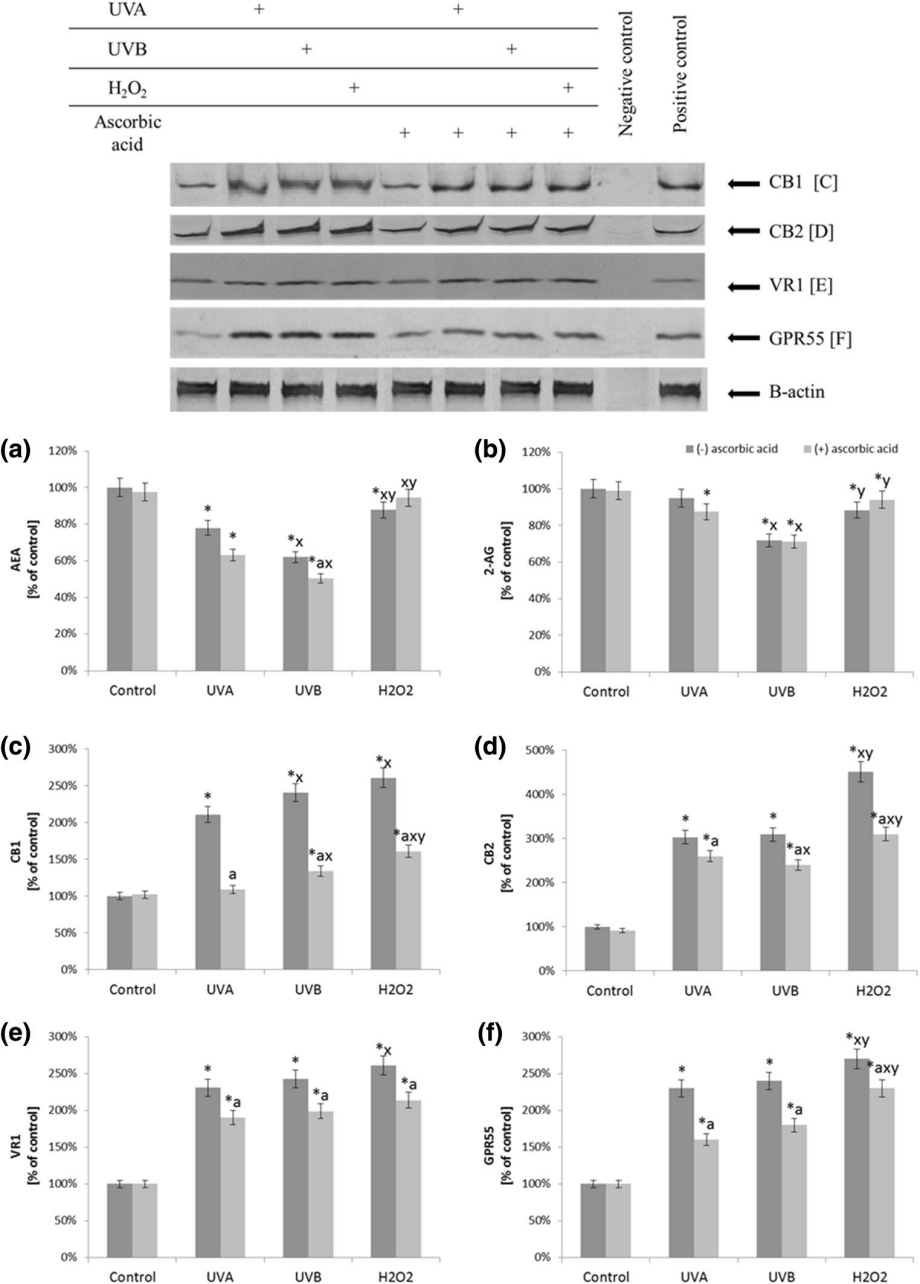
The level of endocannabinoids (AEA and 2-AG) (**a, b**) and the expression of endocannabinoids receptors (CB1, CB2, VR1, GPR55) (**c**–**f**) in fibroblasts after exposure to UVA (20 J/cm^2^), UVB radiation (200 mJ/cm^2^), H_2_O_2_ (200 µM), and ascorbic acid (100 µM) expressed as a percentage of the value of the control cells. Mean values ± SD of three independent experiments are presented. *Statistically significant differences vs. control group, *p* < 0.05; *a* statistically significant differences vs. group without ascorbic acid, *p* < 0.05; *x* statistically significant differences vs. UVA group, *p* < 0.05; *y* statistically significant differences vs. UVB group, *p* < 0.05;

Ascorbic acid also affects changes in level of other lipid mediators—endocannabinoids and their receptors caused by UV radiation or chemical treatment. In fibroblasts, decrease in AEA level after treatment with UVA, UVB and H_2_O_2_ (by 22, 38, and 12% respectively) is observed. Furthermore, the exposure of fibroblasts to UVB radiation and H_2_O_2_ treatment significantly reduces the level of 2-AG (Fig. 6). Simultaneously, there is more than two- and threefold increase in the amount of endocannabinoids receptors (CB1/2, VR1, and GRP55) in the membrane fraction after fibroblasts exposure to UV radiation and H_2_O_2_ treatment. Also, in cells treated with ascorbic acid changes in endocannabinoids receptors compared to cells without rutin treatment are observed. Ascorbic acid causes decrease in all endocannabinoid receptor expression (by approximately 50% in the cause of CB1, and 15–20% in the rest of them) (Fig. 6).

### DNA modifications

Ascorbic acid treatment protects cells against oxidative DNA damage induced by UV and H_2_O_2_. Both UVA and UVB radiation and H_2_O_2_ treatment increased the levels of 8-OHdG by 30, 70, and 110%, respectively. Ascorbic acid treatment protects DNA against H_2_O_2_-induced decrease in 8-OHdG level by approximately 10% (Table 2).

### Protein modifications

Treatment cells with ascorbic acid protects against oxidative damage of protein estimated as carbonyl groups. The results show that after UVA and UVB radiation and H_2_O_2_ treatment, the carbonyl groups levels increased (by approximately 50, 90, and 110%, respectively), while supplementation with ascorbic acid reduced the carbonyl groups levels by approximately 14, 18, and 20% for UVA, UVB, and H_2_O_2_ treatment, respectively (Table 2). Simultaneously, UV radiation, as well as H_2_O_2_ treatment causes also increase in dityrosine level by about 65, 50, and 90%, respectively. Ascorbic acid treatment significantly reduces those effect in the case of UVB radiation and H_2_O_2_ treatment (Table 2).

**Fig. 7:**
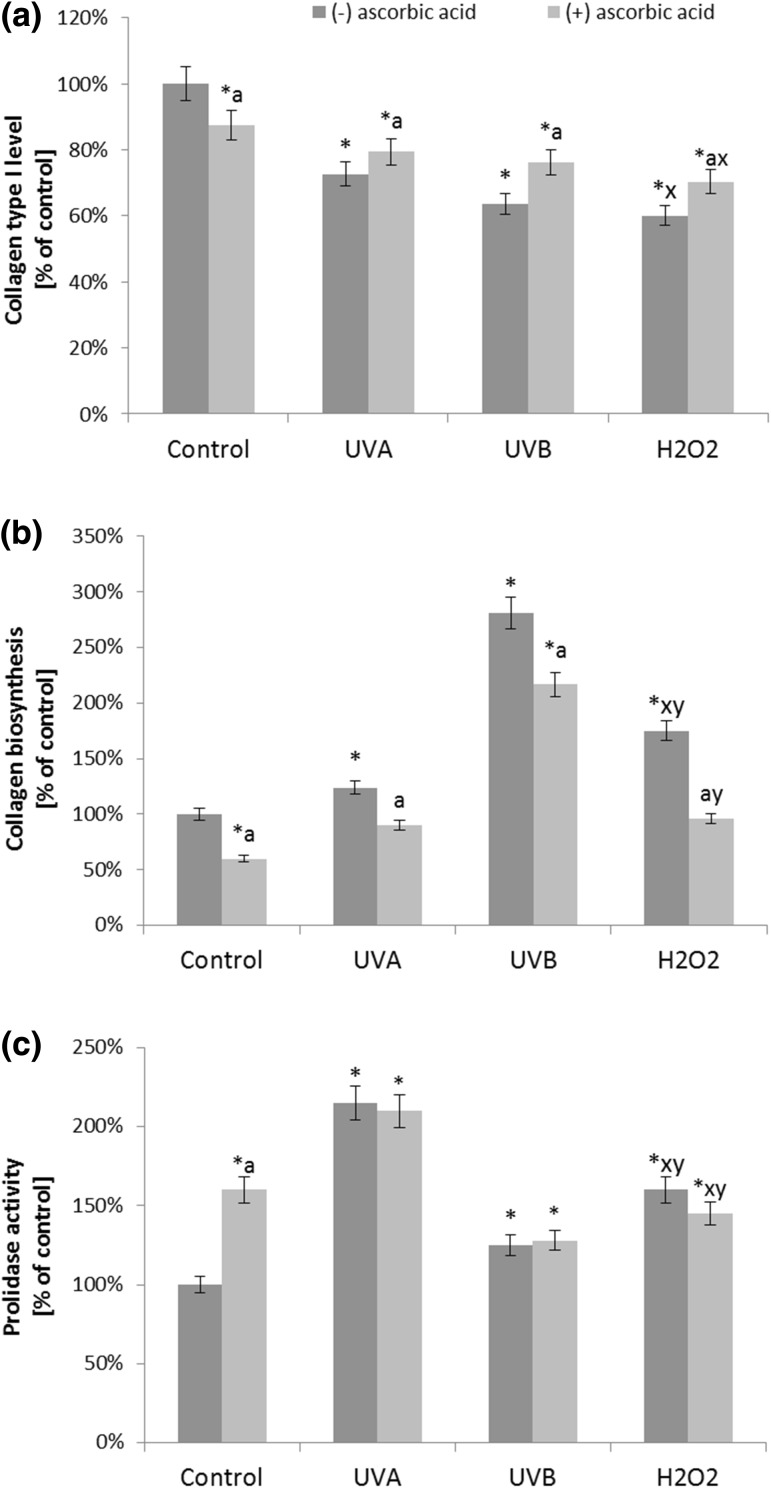
The level of collagen type I expression (**a**), its biosynthesis (**b**) and prolidase activity (**c**) in fibroblasts after exposure to UVA (20 J/cm^2^), UVB radiation (200 mJ/cm^2^), H_2_O_2_ (200 µM), and ascorbic acid (100 µM) expressed as a percentage of the value of the control cells. Mean values ± SD of three independent experiments are presented. *Asterisk* statistically significant differences vs. control group, *p* < 0.05; *a* statistically significant differences vs. group without ascorbic acid, *p* < 0.05; *x* statistically significant differences vs. UVA group, *p* < 0.05; *y* statistically significant differences vs. UVB group, *p* < 0.05

The impairment of the redox status in fibroblasts after UV radiation, as well as treatment with H_2_O_2_, disturbs collagen type I biosynthesis and its metabolism. Supplementation with ascorbic acid in all of the tested groups, significantly increased collagen type I expression (Fig. 5). Changes in the expression of collagen type I may result from enhanced collagen biosynthesis. Treatment with UV radiation and H_2_O_2_ treatment contributed to a decrease in protein biosynthesis by 38, 65, and 53% for UVA, UVB, and H_2_O_2_, respectively, compared to the control cells. Simultaneously, supplementation of the cells with ascorbic acid prevented UV-dependent inhibition of collagen biosynthesis, and under these conditions, collagen was decreased by only 23, 32, and 5% for UVA, UVB, and H_2_O_2_, respectively (Fig. 7). Exposure to UV radiation, as well as treatment with H_2_O_2_, enhanced the activity of prolidase, which is the enzyme involved in the metabolism of collagen by the recovery of proline necessary for its biosynthesis.

Prolidase activity reflects the changes in collagen expression. The cells treated with UVB radiation and H_2_O_2_ evoked the highest decrease in prolidase activity (by approximately 35 and 30%). Ascorbic acid was found to evoke its activity and had a significant protective effect on the prolidase activity under the described conditions (Fig. 7).

### Apoptosis

**Fig. 8:**
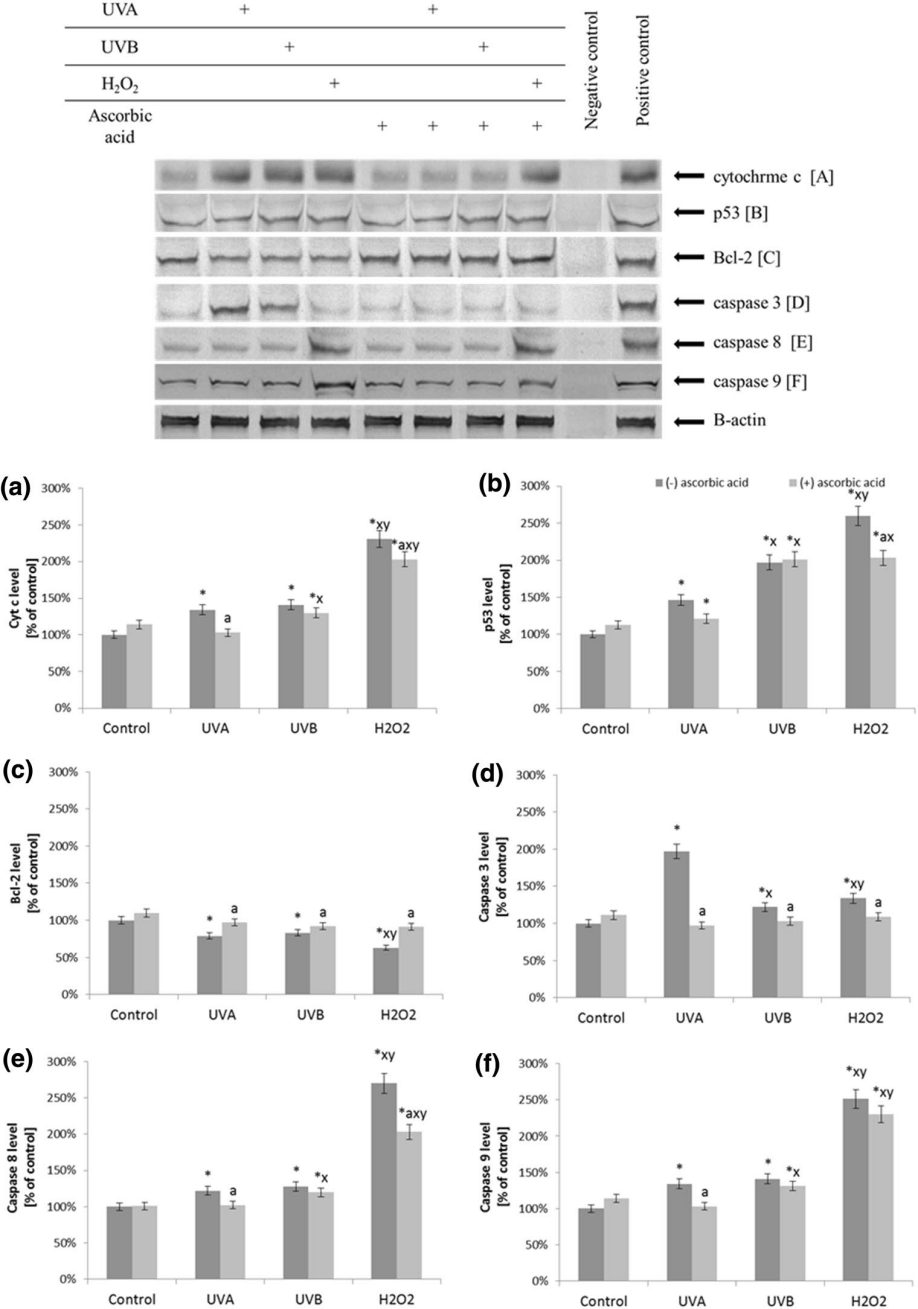
The level of pro- and anti-apoptotic proteins [cytochrome c (**a**), p53 (**b**), Bcl-2 (**c**)], and executive caspases [3, 8, and 9 (**d**–**f**)] in fibroblasts after exposure to UVA (20 J/cm^2^), UVB radiation (200 mJ/cm^2^), H_2_O_2_ (200 µM), and ascorbic acid (100 µM) expressed as a percentage of the value of the control cells. Mean values ± SD of three independent experiments are presented. *Asterisk* statistically significant differences vs. control group, *p* < 0.05; *a* statistically significant differences vs. group without ascorbic acid, *p* < 0.05; *x* statistically significant differences vs. UVA group, *p* < 0.05; *y* statistically significant differences vs. UVB group, *p* < 0.05

Ascorbic acid affects the expression levels of proteins involved in apoptosis. UV radiation and treatment with H_2_O_2_ reduced the levels of anti-apoptotic protein Bcl-2 by 30 and 40%, respectively, what is abolished by ascorbic acid treatment. In addition, increased levels of cytochrome c, p53, caspase-3, caspase-8, and caspase-9 were observed. However, ascorbic acid treatment caused slight decreases in the levels of these proapoptotical proteins, especially caspase-3 (Fig. 8).

## Discussion

Everyday exposure of the human body to external environmental factors (physical and chemical) causes metabolic disturbances in the cells of various tissues. The most affected tissue is the skin, especially its living cells such as fibroblasts, which have the ability to constantly proliferate. The consequences of skin exposure to UV radiation or chemicals are well recognized as a perturbation in the immune system and redox balance resulting in skin aging, as well as the promotion of serious diseases, such as skin cancers [45]. Fibroblasts exposed to highly energetic and strongly cytotoxic and mutagenic UVB irradiation, as well as to less energetic but more penetrating UVA radiation react in different ways to both types of radiation [46, 47]. Hydrogen peroxide, which belongs to ROS, causes various dose-dependent responses in fibroblasts [48]. However, both above physical and chemical factors develop oxidative stress in the cells [46, 49].

The data presented in this study show that the most important cytoplasmic enzymes responsible for the generation of superoxide anion, the first of all radicals, xanthine and NADPH oxidases, are strongly activated by UV irradiation, UVB in particular and by hydrogen peroxide. Therefore, significantly enhanced level of superoxide anion is observed and the chain of free radical reactions is initiated, particularly that activity of Cu,Zn-SOD—the enzyme responsible for superoxide anion dismutation is significantly decreased. The oxidative potency of hydrogen peroxide is similar to UVB and significantly stronger than UVA radiation. It is accompanied by a decreased level of endocannabinoids (AEA and 2-AG), which was shown to suppress ROS and inflammatory response [50], e.g., anandamide inhibiting NF*κ*B activation by interfering with phosphorylation of I*κ*B*α* [15]. It has been demonstrated that hydrogen peroxide significantly weaker diminishes anandamide level that may result from significantly enhanced lipid peroxidation (estimated by isoprostanes and MDA level) that is accompanied by activation of phospholipases that participate in endocannabinoids synthesis. Higher ROS generation as well as enhanced inflammatory response estimated by NF*κ*B expression and upregulation of the TNF*α*—NF*κ*B target protein may be connected with significant upregulation of the examined cannabinoid receptors, particularly CB1, which predispose cells to enhanced inflammation [51]. Upregulation of CB1/2 receptors were also observed in gingival fibroblasts under pathological conditions [52]. Thus, taken together, regulatory properties of endocannabinoid system components may either add or synergize fibroblasts protective activity.

Ascorbic acid added to medium of UV irradiated and hydrogen peroxide treated fibroblast similarly as other antioxidants diminished examined oxidases activity which resulted in a decrease in superoxide generation and inflammation response through NF*κ*B inhibition. Moreover, ascorbic acid particularly strongly prevented activation of CB1 receptor that is involved in ROS and pro-inflammatory cytokines generation [53]. The most striking chemical property of ascorbic acid is its ability to act as a reducing agent, which very effectively quenches free radicals [4], and therefore protects the skin against long-lived free radicals. Donation of one electron by ascorbate to reactive radicals makes the ascorbyl radical much less reactive than others, which undergoes a disproportionation reaction, regenerating some ascorbate [54]. Therefore, ascorbic acid decreasing ROS level prevents oxidative modifications caused by ROS, which, in this study, occurred after UV irradiation and hydrogen peroxide treatment. It is particularly visible in the case of phospholipid peroxidation. The main constituents of membrane phospholipid are polyunsaturated fatty acids (PUFA’s) particularly sensitive to ROS-mediated oxidative modifications what leads to the generation of chemically independent products formed by cyclization, as well as the fragmentation of PUFAs [55]. Hydrogen peroxide particularly enhanced reactions of cyclization with generation of 8-isoprostanes. Ascorbic acid did not prevent this reactions. UV radiation and, to a greater degree, chemical initiation of lipid peroxidation caused significantly increase in the levels of MDA and 4-HNE, the main products of PUFAs fragmentation and ascorbic acid only partially decreased the level of these reactive aldehydes. It is known that electrophilic aldehydes may further form adducts with the reactive groups of protein, DNA, or lipids, what modifies their functions [56]. They react with protein amino acids residues, such as cysteine, histidine and lysine residues *via* Michael addition and with lysine generated Schiff base and form relatively stable and hardly metabolized protein adducts [57]. By changing the structure and function of diverse structural and regulatory proteins, 4-HNE achieves not only cytotoxic, but also regulatory functions in various pathophysiological processes [58].The formation of 4-HNE-protein adducts enables 4-HNE to participate in multi-step regulation of cellular metabolic pathways, as well as antioxidant enzymes activity [59]. Moreover, 4-HNE is considered to act as a second messenger of free radicals and as the major bioactive marker of lipid peroxidation [60]. Increase in 4-HNE suggests systemic oxidative stress characteristic for development of skin diseases like rosacea, psoriasis, or skin cancer transformation [61–63]. However, showed in this work ascorbic acid induced decrease in 4-HNE also indicates ascorbic acid protective effect on the skin cells during stress conditions as well as physiologic aging [64].

Fibroblasts DNA modifications (8-OHdG) level particularly after hydrogen peroxide treatment is similarly as MDA and carbonyl groups twice higher than in control. The above results are accompanied by the reduced level of all of the non-enzymatic antioxidants what does not allow them to act efficiently and protect cellular components against oxidation. Changes in the amount and structure of the membrane lipids is certainly important for predefining the risk of such a lipid peroxidation process that may be further triggered and intensified by a decrease in vitamin E, which physiologically cooperates with GSH in phospholipid protection [37]. However, the level of GSH is significantly decreased after physical and chemical fibroblasts treatment which is probably connected with strong and partially irreversible modifications of its structure, caused by ROS or reactive aldehydes, because the activity of GSSG-R, the enzyme regenerating GSH from its oxidized form is significantly enhanced. Ascorbic acid that partially prevents phospholipid does not completely prevent GSH against oxidation what indicates that this tripeptide is modified by ROS as well as by electrophilic aldehydes similarly as other polypeptides [65]. This situation favours a lack of total renewal of vitamin E and A, which are the main lipophilic antioxidants actively protecting phospholipid. However, after ascorbic acid treatment cooperation between GSH and lipophilic antioxidants is more effective and the level of lipid peroxidation products is rather lower. The maintenance of the membrane phospholipid structure is controlled by the combined action of non-enzymatic antioxidants present in the cells, but in particular, by GSH-Px, the action of which is exhibited toward peroxides, particularly lipid peroxides. The results of this paper show that both physical and chemical factors enhance GSH-Px activity, but the efficacy of its action may not be increased; conversely, the action of GSH-Px in fibroblasts is decreased because of a diminished level of reduced glutathione, which is a co-substrate of this enzyme and limits its action [66]. However, treatment with ascorbic acid partially enhances the level of GSH and allows GSH-Px to act more effectively and help lipophilic non-enzymatic antioxidants protect phospholipid parts of the membranes. However, not only these cellular components are damaged by oxidative conditions. The protein structure is also modified directly by the action of ROS or electrophilic aldehydes [67]. Damage to the protein structure after UV irradiation, as well as hydrogen peroxide treatment, was confirmed by an increase in protein carbonyl groups and dityrosine level. The observed changes in the protein structure may lead to functional disturbances, e.g., the decrease in Cu,Zn-SOD activity after fibroblasts are treated chemically and physically. Treatment with ascorbic acid causing decrease in ROS generation as well as more efficient antioxidant protection partially prevents protein molecules against oxidative modifications.

However, antioxidant defence mechanisms in fibroblasts UV irradiated and treated with hydrogen peroxide is disturbed not only at molecular level but also at transcriptional level that is connected with regulation of cytoprotective protein genes, e.g., phase II enzymes [68]. The main regulator of antioxidant protein expression is the transcription factor Nrf2, which is regulated by redox conditions [69]. Therefore, after UV irradiation, as well as hydrogen peroxide addition, we also found significantly higher levels of phosphorylated Nrf2, which is considered to be more active than its unphosphorylated form [70]. Under physiological conditions, cytoplasmic Nrf2 is binding by Keap1 to regulate degradation [71], but electrophilic aldehydes, mainly 4-HNE, generated after UV irradiation as well as hydrogen peroxide treatment may lead to the oxidation of Keap1 cysteine catalytic residue, causing a lack of binding and/or the dissociation of Nrf2 from the complex, which results in its translocation into the nucleus. The critical Keap1 cysteine residues Cys273/288 are particularly susceptible to lipid peroxidation products including electrophilic aldehydes as well as prostaglandin derivatives as was earlier shown [72]. Ascorbic acid, through a reduction of phospho-Nrf2 levels, limits the translocation of this factor to the nucleus and its cytoprotective action because the added antioxidant takes over part of the protective activity. Moreover, these results show that ascorbic acid through a significant decrease in the expression of cannabinoid receptors in fibroblasts after UV irradiation/H_2_O_2_ treatment may diminish signal transduction through the decreases in Nrf2 phosphorylation. However, Nrf2 expression is correlated with another transcription factor, NF*κ*B, because both of them are activated under oxidative conditions. It was earlier shown that NF*κ*B subunits induce the transcription of Nrf2 in the human leukemia cells at a specific promoter *κ*B site [73]. It was confirmed in this study that such induction concerns similarly chemical and physical factors action on fibroblasts. Antioxidant treatment inhibits activation of NF*κ*B so ascorbic acid administration led to lower NF*κ*B expression as well as Nrf2.

Independently of Nrf2 level its action is regulated by series of activators and inhibitors. The present study has also showed that UV radiation, as well as H_2_O_2_ treatment enhanced the expression of KAP1 that is an inhibitor of the Nrf2-Keap1-Cul3 complex formation, which was attenuated non-significantly by ascorbic acid treatment. Moreover, it was previously shown in HEK293 cells that decreases in KAP1 expression caused by gene knockdown led to disruption in KAP1-mediated transcriptional repression of other Nrf2 activator—p21 [74]. These facts suggest that observed in this paper antioxidant capacity of ascorbic acid decreases the Nrf2 activity by KAP1-dependent pathway suppressing, particularly after UV irradiation.

Free and active Nrf2 is translocated to the nucleus and bonded to the DNA at ARE sequences [75]. This interaction is facilitated by UV-induced as well as hydrogen peroxide reduction in Bach1 level, which also binds DNA sequence within the ARE. In the context of UVA-induced oxidative stress, it is known that Bach1 transcript level is reduced [76], but redox-mediated regulation of Bach1 is an alternative mechanism to induce multiple ARE-dependent genes [77]. Moreover, reactive electrophiles, such as 4-HNE whose increase is observed after UV irradiation and H_2_O_2_ treatment, may enhance inactivation of this transcription protein induction to cell protection against oxidative stress what is observed in this study may also inactivate Bach1 as an additional cytoprotective response. However, cells treatment with ascorbic acid, particularly after chemical oxidation, prevented oxidation of Bach1 cysteine residues, thereby enhancing Bach1 biological activity.

Moreover, our observations indicate that lower endocannabinoids levels, after stress induced by UV and hydrogen peroxide, promote NF*κ*B expression and that this effect is not mediated by their interactions with receptors (CB1/2, VR1 and GPR55) because their activation was opposite to changes in endocannabinoids level. However, changes in NF*κ*B expression might underlie anti-inflammatory and proapoptotic effect of AEA [78]. Therefore, relatively high level of AEA and activation of CB1 receptor as well as the enhanced level of p53 releasing of cytochrome c from fibroblasts mitochondria after chemical treatment may explain proapoptotic activity of hydrogen peroxide in particular, with intrinsic pathway activation. Ascorbic acid partially reduced CB1 activation as well as protected fibroblasts from chemically or physically induced apoptosis. The obtained data also suggest that ascorbic acid through changes in the endocannabinoid system response as well as inhibition of HO-1 expression may modulate fibroblasts survival. However, its protective abilities against oxidative damages of macromolecules are insufficient. That is confirmed by only partial reduction of p53, a protein involved in activation of DNA repair mechanisms and induction of apoptosis in response to DNA damage. Therefore, oxidative damages of DNA, but also lipid and protein levels after ascorbic acid treatment are still significant what indicates for these macromolecules functional disturbances.

Ascorbic acid is also involved in the prevention of chemically and physically induced apoptosis pathway through a significant but only partial decrease in TNF*α* level as well as expression of initiator caspases, caspases-8 and -9, but completely normalized expression of executioner caspase-3. One of the modulators of apoptosis is 4-HNE that can stimulate intrinsic and extrinsic apoptotic pathways and interact with typical factors such as tumor protein 53, JNK, Fas or mitochondrial regulators, but also is involved in activation of caspases 9 and 3 [79, 80]. Since ascorbic acid does not completely prevents 4-HNE increase under the influence of chemical and physical factors, it may be indicated as a responsible for sustained elevated levels of certain factors associated with the apoptotic process.

Moreover, changes in the level of the main skin protein—collagen is observed. Higher level of NF*κ*B promotes a decrease in collagen biosynthesis at the transcriptional level, because this transcription factor is a well-recognized inhibitor of the expression of a1 and a2 subunits of type I collagen [81, 82]. However, these studies show that the mechanism responsible for the biosynthesis of collagen in the human skin fibroblasts treated with the studied stressed factors may occur also in another ways. The observed oxidative stress contributes to the decrease in collagen level, because ROS can also affect its proper biosynthesis [66, 83]. On the other hand, prolidase is also responsible for proline providing for collagen synthesis and may regulate its turnover as well as be a rate-limiting factor in the regulation of collagen production [84]. Our study shows enhanced prolidase activity in the fibroblasts following treatment with UV radiation and H_2_O_2_, however, the level of collagen is diminished what is parallel with a decrease in the level of ascorbate that is required as a cofactor for prolidase action [85]. Collagen synthesized in the absence of ascorbic acid is insufficiently hydroxylated and does not form fibers properly, giving rise to poor wound healing [86]. Therefore, ascorbic acid added to fibroblasts medium promotes synthesis and proper structure of collagen. Enhanced prolidase activity was found during the fibrotic process [87]. Moreover, collagen production and prolidase activity were found to correlate with each other in cultured human skin fibroblasts treated with anti-inflammatory drugs or during the experimental aging of these cells [88].

## Conclusion

**Fig. 9:**
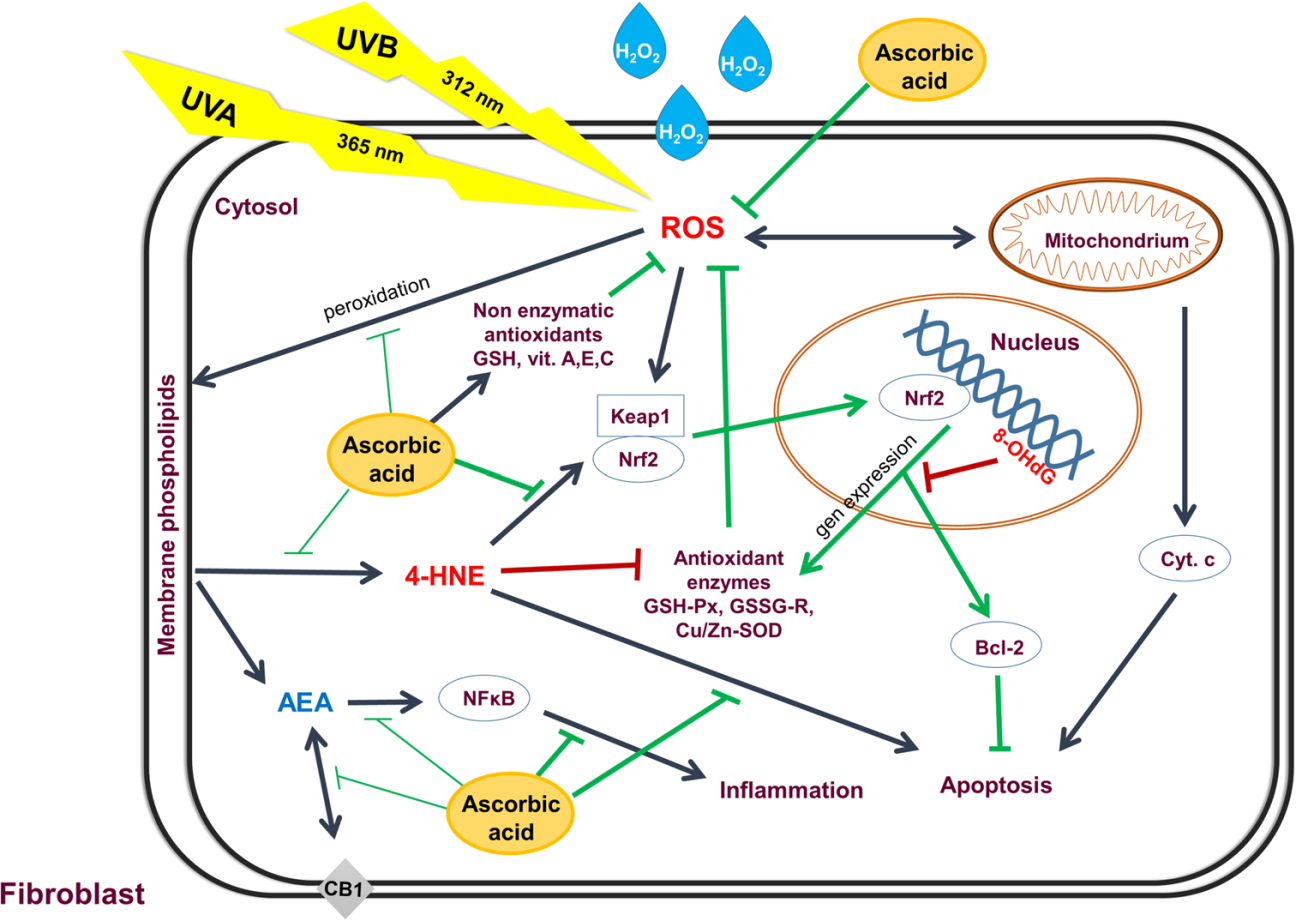
A scheme summarizing the ascorbic acid effect on ROS generation, lipid mediators and its consequences for the redox homeostasis in fibroblasts following exposure to UVA and UVB radiation, as well as H_2_O_2_ treatment

Ascorbic acid partially prevents redox imbalance in fibroblasts, caused by hydrogen peroxide and UV irradiation, but as a Nrf2/ARE pathway inhibitor rather weakly protects cellular macromolecules against damages that contribute to an increase in the level of lipid mediators such as lipid peroxidation products and endocannabinoids participating in different metabolic pathways including apoptosis (Fig. 9) [89].
